# Occupational Heat Stress Profiles in Selected Workplaces in India

**DOI:** 10.3390/ijerph13010089

**Published:** 2015-12-29

**Authors:** Vidhya Venugopal, Jeremiah S. Chinnadurai, Rebekah A. I. Lucas, Tord Kjellstrom

**Affiliations:** 1 Department of Environmental Health Engineering, No.1, Ramachandra Nagar Porur, Chennai, Tamilnadu 600116, India; jeremiah@ehe.org.in; 2 Department of Public Health and Clinical Medicine, Epidemiology and Global Health, Umeåa University, 901 87 Umeå, Sweden; r.a.i.iucas@bham.ac.uk (R.A.I.L.); kjellstromt@yahoo.com (T.K.); 3 School of Sport, Exercise and Rehabilitation Sciences, The University of Birmingham Edgbaston, Birmingham B15 2TT, UK; 4 Health and Environment International Trust, 168 Stafford Drive, Mapua 7005, Nelson, New Zealand

**Keywords:** occupational heat stress, health impacts, perception, prevention

## Abstract

Health and productivity impacts from occupational heat stress have significant ramifications for the large workforce of India. This study profiled occupational heat stress impacts on the health and productivity of workers in select organized and unorganized Indian work sectors. During hotter and cooler seasons, Wet Bulb Globe Temperatures (WBGT) were used to quantify the risk of heat stress, according to *International workplace* guidelines. Questionnaires assessed workers’ perceived health and productivity impacts from heat stress. A total of 442 workers from 18 Indian workplaces participated (22% and 78% from the organized and unorganized sector, respectively). Overall 82% and 42% of workers were exposed to higher than recommended WBGT during hotter and cooler periods, respectively. Workers with heavy workloads reported more heat-related health issues (chi square = 23.67, *p* ≤ 0.001) and reduced productivity (chi square = 15.82, *p* ≤ 0.001), especially the outdoor workers. Heat-rashes, dehydration, heat-syncope and urinogenital symptoms were self-reported health issues. Cited reasons for productivity losses were: extended-work hours due to fatigue/exhaustion, sickness/hospitalization and wages lost. Reducing workplace heat stress will benefit industries and workers via improving worker health and productivity. Adaptation and mitigation measures to tackle heat stress are imperative to protect the present and future workforce as climate change progresses.

## 1. Introduction

Climate change poses a serious threat to the health of the general population and adds to the risk of occupational heat stress, which has significant ramifications for the large workforce employed in diverse industrial settings [1,2]. Predicted changes in air temperatures and/or humidity as part of local climate change have been forecasted to cause occupational health and work productivity concerns [3,4,5]. Bennet and McMichael [6] have also highlighted the negative impacts of non-heat related climate change effects on occupational health and safety. However, there remains a significant knowledge gap concerning the thermal work conditions and related health impacts of some occupations, particularly those occupations carried out by low socioeconomic groups in tropical climates. In order to better understand the impacts of climate change on occupational health and productivity, it is imperative that current work environments are assessed to identify the occupational settings and working population most vulnerable to increasing global temperatures.

Climate change directly increases occupational heat stress for workers, which impacts their health and productivity [5,7,8]. Heat stress is a physical hazard and potential health risk that can lead to a range of conditions from discomfort, headache, syncope, progressive loss of mental alertness, heat stroke and even death in extreme cases [4]. In developed countries, the negative relationship between excessive heat stress and occupational injury is well recognised [9,10,11,12]. Despite this, heat stress remains an unattended occupational health problem with dire consequences [13,14]. The risks of excessive heat exposure are greater still in tropical developing countries where large work forces perform manual and heavy labour for long periods under very hot and humid conditions with minimal access to cooling interventions [4,15]. Workers exposed to such conditions are highly vulnerable to occupational injury and heat related illnesses [16,17], especially outdoor workers with heavy workload and direct sun exposure [14]. Furthermore, it becomes progressively harder to work productively in very hot and humid tropical conditions [18].

Regional and global assessments of the impacts of occupational heat exposures on health and productivity has begun in recent years. In Central America and South-East Asia, research has demonstrated that workers in different trades are exposed to excess levels of heat stress with resultant reductions in production and/or serious health consequences [3,16]. To date, few studies have examined heat related health problems among the working population in India. A study investigating an Indian glass manufacturing unit suggested the need for improved working conditions and revised standards considering the high ambient working temperatures [19]. Somnathan *et al.* reported reduced productivity with higher ambient temperatures in Indian manufacturing industries [20]. We have also reported health and productivity losses in construction workers due to occupational heat exposure [21] as well as the need for stronger public health and labourpolicies and reforms to protect the large working population engaged in construction in developing countries like India [22].

India is a very large country with a diverse and often extreme climate [23]. Additionally, India has a very large unorganized work sector, and outdoor work is very common in both organized and unorganized sectors. There remain challenges and opportunities in occupational health services in India, with improvements needed in occupational health, hygiene and safety services in the organized sector as well as the need for some basic occupational health protection (currently non-existent or very limited) in the unorganized sector [24]. Therefore, the aim of the current study was to profile occupational heat stress and its impact on the health and productivity of workers from a range of workplaces from both the organized and unorganized work sectors in India during hotter and cooler seasons.

## 2. Experiment Design

Occupational heat stress and workers’ perceptions of the impact of heat on their health and productivity was studied in 18 different workplaces. Workplaces were located in four cities of India (Chennai and Tiruchirapalli in Tamilnadu, Bengaluru in Karnataka and Mumbai in Maharastra). Data collection was conducted in each workplace for two seasons, once during the “hotter season” (April–June) and another during the “cooler season” (November–January), to estimate the impacts of occupational heat stress impacts on workers during two distinct seasons. Prior ethical clearance from the Institutional Ethics Committee (IEC) and permission from the concerned management was obtained for the study. The risks and benefits of participating in the study were explained to the workers and signed informed consent was obtained. A walk-through audit was done in each workplace to identify sampling locations for heat monitoring and to make observations about the workplace ventilation and existing cooling provisions. Both qualitative and quantitative data were collected on different days when work was in progress. Though workplaces were visited during two seasons, the qualitative assessments using questionnaires (Supplement Information) were not necessarily administered to the same participants in both seasons.

### 2.1. Quantitative Assessments of Heat and Work Intensity

Quantitative data on heat stress exposure was assessed via measurements of the Wet Bulb Globe Temperature (WBGT) and using a portable heat stress monitor, (QuesTemp 34; QUEST Technologies, Oconomowoc, WI, USA), which has an accuracy level of ±0.5 °C between 0 °C and 120 °C of dry bulb temperature and ±5% relative humidity (RH) between 20% and 95% RH. The WBGT combines the effect of the four main thermal components affecting heat stress: air temperature, humidity, air velocity and radiation, as measured by the dry bulb, wet bulb and globe temperatures. Globally, the WBGT index is the most commonly used heat index in heat stress assessments [3,25,26,27,28] and is used by many international organizations for defining heat exposure thresholds or limits for workers [26,29]. Although there are disputed limitations with the WBGT index (*i.e*., inadequate response to humidity and air movement) and associated guidelines (*i.e*., too conservative) [30] it remains a simple and valid method of determining heat stress [31]. Ambient WBGT measurements were made during normal working hours, between 10 a.m.–4 p.m. in most of the workplaces, except in brick industry, where the measurements were made at 4a.m. in the morning. The QuesTemp was calibrated at the start and end of a measurement day. For measurements, the QuesTemp was placed at a height of 3.5 feet (1.1 m) for standing individuals and 2 feet (0.6 m) for seated individuals. A tripod mounting was used to get the instrument away from anything that might block radiant heat or flow and workers were asked to stand away from the instrument to minimize variations in temperature and radiant heat. The equipment was allowed to stabilize for about 15 min before taking the measurements in each location. The number of locations WBGT measurements was taken in each workplace varied depending on the work intensities of the workers and potential heat exposure zones in the workplace. The number of days the assessments were conducted in each workplace also varied with each workplace which ranged between one to three successive days in each work sector. The minimum, maximum and standard deviation of WBGT values from each workplace was computed from the total number of ambient heat stress measurements taken at various locations in the each workplace. Workers’ work intensity was judged by a trained Industrial Hygienist according to *American Conference of Governmental Industrial Hygienists *(ACGIH) guidelines for evaluating Metabolic Rate Categories and the Representative Metabolic Rate with Example Activities [32]. ACGIH WBGT permissible heat exposure threshold limit values (TLV) were also used to evaluate the risk of heat stress and the corresponding WBGT under which continuous work during an hour could be safely undertaken [3]. Workers in evaluated areas wore work clothes (long sleeve shirt and pants) or cloth (woven material). Accordingly, no WBGT correction factor for clothing was used in the current study [32].

### 2.2. Qualitative Assessment of Heat Perception and Impacts

Qualitative data about the perceptions on heat exposures, clothing acclimatization, symptoms of potential health impacts, productivity losses and coping mechanisms were collected by administering a modified version of HOTHAPS questionnaire (See Supplement Information [33]). This questionnaire has seven parts: (1) general information; (2) type of work (as per ACGIH, 2010); (3) workers’ exposure to heat; (4) health impacts; (5) productivity impacts; (6) impacts of clothing and (7) coping mechanisms. The HOTHAPS questionnaire was administered to the workers who volunteered for the study in various locations within each workplace. Any vague answers due to ambiguity in understanding the question was clarified by the trained interviewer. Question pertaining to impacts of heat stress was very clearly directed towards the season in which the questions were administered. Responses concerning health and productivity were specifically related to workplace heat stress. An elaborate section on self-reported heat related health illnesses was administered and the symptoms of each illness were explained to the study participant by the interviewer. A person’s health was considered as affected by heat stress if he/she experienced one of the following heat related symptoms at work *i.e.*, excessive sweating, excessive thirst, tiredness, cramps, headache, nausea/vomiting, fainting or prickly heat. Productivity loss due to heat stress was defined as loss in production, or not achieving set work targets, or loss workdays/work hours due to fatigue/exhaustion, or sickness/hospitalization, and/or wages lost due to heat or heat related illnesses. Major observations were made by the investigators and the workers about the workplace conditions.

### 2.3. Data Analysis

All data analysis was done using Microsoft Excel 2007 and R-statistical software. Single proportions were tested for significance using the *Z*-test. Bivariate analysis was done for identifying associations using chi square test. The crude Odds Ratios (OR) is presented as the measure of association, the cutoff of 0.05 was used to interpret the significance of the *p*-values for all analysis. Multivariate logistic regression analysis using stepwise method was done for controlling possible confounders. The adjusted ORs thus calculated are presented with the corresponding *p*-values and 95% CIs. Chi square test was used to compare seasonal differences in qualitative assessments of heat and productivity for workers assessed in both hotter and cooler seasons.

## 3. Results

### 3.1. Study Population

A total of 18 workplaces were evaluated and 442 workers (238 in hotter and 204 in cooler seasons) were interviewed, of whom 71% (*n* = 314) were males and 29% (*n* = 128) were females. The mean age of the study population was 35.8 ± 12.7 years and approximately half of the workers (*n* = 248) had some basic level of education. 75% of the study populations were non-smokers, 24% of the population consumed alcohol and about 28% had some pre-existing medical condition such as diabetes or hypertension. Repeat qualitative measures were able to be gathered from 72 workers in both hotter and cooler seasons.

### 3.2. Heat Stress Profile

**Figure 1:**
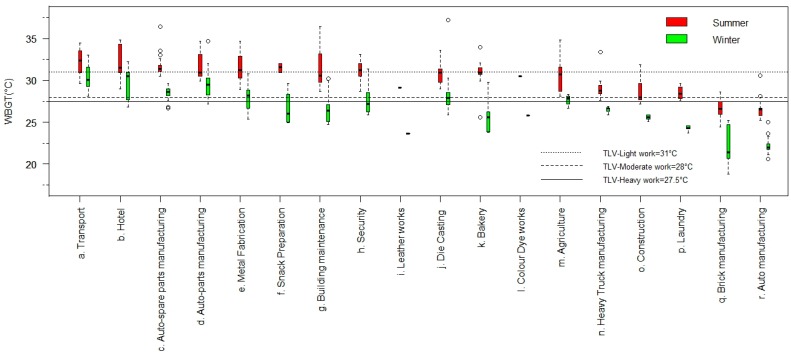
Wet bulb globe temperature **(**WBGT) profiles across various workplaces during cooler (2012) and hotter (2013) seasons in India. The three lines across the graph represent the Threshold Limit Values (TLV) for each of the work categories (31 °C for light work, 28 °C for moderate and 27.5 °C for heavy work categories).

Of the total workers examined in the current study, 71% of workers had direct heat exposures that included furnaces, hot processes and/or direct sun. Hotter and cooler season WBGT profiles along with ambient temperature from the 18 examined workplaces are shown in Table 1 and WBGT profiles alone are illustrated in Figure 1. In the hotter season, 82% of the workers were exposed to WBGTs higher than the recommended TLV as per ACGIH guidelines (Table 2). In cooler seasons, 42% of workers engaged in moderate and heavy work were exposed to higher than recommended WBGT levels. 247 workers were classified as engaged in heavy work, while 186 workers were engaged in moderate work and nine workers were engaged in light work during the hotter and cooler seasons (Table 2).

Across all work intensity categories, workers worked beyond the recommended TLV levels (64%, *p* < 0.001) irrespective of the season. Workers with heavy workloads (*n* = 190, 43% for 2 seasons) were exposed to higher occupational heat stress as compared to the other work categories (Chi square = 22.32, *p* ≤ 0.001). Workers with heavy workload had higher odds of developing heat-related health problems compared to workers with moderate workload (OR = 0.070, *p* = 0.013) and light workloads (*p* = 0.001).

**Table 1:** Seasonal exposure profiles for occupational heat stress for different workplaces in India (2012–2013).

Sl. No	Sectors	Type	Hotter Season (April–June)					Cooler Season (November–January)				
			Ambient		WBGT °C			Ambient		WBGT °C		
			DB *(°C)	RH *(%)	Min	Max	Avg ± SD	DB *(°C)	RH *(%)	Min	Max	Avg ± SD
1.	Transport (#M = 11)	Indoor	32.7	60.3	29.6	34.5	32.2± 1.8	30.5	67.3	28.1	33.0	30.4 ± 1.5
2.	Hotel (#M = 8)	Indoor	35.3	45.6	29	34.8	31.9 ±2.7	28.9	55.0	26.8	32.2	29.5 ± 2.1
3.	Auto spare parts (#M = 19)	Indoor	35.9	40.0	30.5	36.4	31.8±1.3	31.8	53.0	26.7	29.6	28.5 ± 0.9
4.	Auto-parts (#M = 25)	Indoor	32.9	58.0	29.9	34.7	31.7±1.6	27.5	75.4	27.2	34.7	29.6 ± 1.6
5.	Metal Fabrication (#M = 24)	Outdoor	36.9	39.5	28.9	34.7	31.5 ±1.6	29.5	55.0	25.4	30.8	28.0 ± 1.6
6.	Snacks preparation (#M = 5)	Indoor	33.8	49.7	30.9	32.0	31.5 ±0.5	29.4	32.4	24.9	29.6	26.7 ± 2.9
7.	Building Maintenance (#M = 20)	Outdoor	37.1	39.9	28.7	36.4	31.4 ±2.2	29.8	51.9	24.7	30.2	26.8 ± 1.9
8.	Security (#M = 20)	Outdoor	36.2	41.2	28.7	33.1	31.1 ±1.2	29.3	64.3	25.9	31.4	27.7 ± 1.8
9.	Leather works (#M = 1)	Indoor	34.0	52.0	31.0	ND	31.0	29.0	48.0	23.6	ND	23.6
10.	Die casting (#M = 18)	Indoor	33.9	44.4	29.0	33.6	30.9 ±1.4	29.7	63.1	25.9	37.2	28.1± 2.3
11.	Bakery (#M = 9)	Indoor	35.0	50.7	25.6	34.0	30.8±2.1	29.0	48.6	23.8	29.8	25.6 ± 2.0
12.	Colour dye works (#M = 1)	Indoor	35.0	46.5	30.5	ND	30.5	29.5	43.5	25.8	ND	25.8
13.	Agriculture (#M = 10)	Outdoor	34.4	41.9	28.1	34.8	30.5± 1.9	28.9	62.0	26.7	28.3	27.7 ± 0.7
14.	Heavy Truck (#M = 17)	Indoor	33.6	45.8	27.6	33.4	29.0 ±1.2	29.5	71.6	25.9	26.9	26.5 ± 0.3
15.	Construction (#M = 12)	Outdoor	34.5	38.9	27.2	31.9	28.7 ±1.6	30.0	53.7	25.1	25.9	25.6 ± 0.8
16.	Laundry (#M = 13)	Indoor	32.7	47.5	27.6	29.6	28.5 ±0.8	29.6	62.3	23.7	24.6	24.3 ± 0.3
17.	Brick**^‡^** (#M = 12)	Outdoor	29.1	39.5	24.4	28.6	26.7 ±1.1	20.8	85.0	18.8	25.2	22.2 ± 2.3
18.	Auto (#M = 28)	Indoor	31.5	36.6	25.2	30.6	26.5±1.0	30.7	47.0	20.6	25.0	22.2 ± 0.9

DB, Dry Bulb; RH, Relative Humidity; ND, Not determined; WBGT, Wet Bulb Globe Temperature, #M = number of heat stress measurement locations within a workplace; ‡, Measurements taken early morning (5:00 a.m.–9:00 a.m.) to coincide with workers work schedule; * Average ambient parameters during the time of workplace heat measurements.

**Table 2:** Seasonal distribution of workers exceeding ACGIH recommended threshold limit values in various work intensity categories.

Work Category	Hot Season		Cooler Season		Both Seasons Combined	
	Total Number of Workers	Workers Exceeding TLV	Total Number of Workers	Workers Exceeding TLV	Total Number of Workers	Workers Exceeding TLV
Heavy	132	125 (95%)	115	65 (57%)	247	190 (77%)
Moderate	101	70 (69%)	85	22 (26%)	186	92 (49%)
Light	5	0 (0%)	4	0 (0%)	9	0%
All work categories combined	238	195 (82%)	204	87 (42%)	442	282 (64%)

ACGIH, *American Conference of Governmental Industrial Hygienists*; TLV, Threshold Limit Values.

### 3.3. Heat-Related Health Impacts

Of the 282 workers exposed to excessive occupational heat levels during hotter (*n* = 195) and cooler (*n* = 87) months, significantly more males (*n* = 212, 75%, *p* = 0.005) were exposed compared to the females (*n* = 70, 24.8%) as males had a higher share of heavy labor in almost all the workplaces. Though there are some control interventions in the organized sectors to minimize workers’ heat exposures, in this study no significant difference in heat stress exposure was observed between organized (*n* = 252, 63%) and unorganized sectors (*n* = 190, 65%).Workers who described the nature of their work as being heavy were exposed to significantly higher heat stress levels (*n* = 190, 67%) as compared to workers who described it as moderate (*n* = 186, 33%; chi square = 22.322, *p* ≤ 0.001).

Table 3 reflects the perceptions of workers on heat related symptoms that could potentially impact heir health and productivities resulting from heat stress. The results of the 442 workers’ self-reported affirmative response for any one of the heat related symptoms such as excessive sweating or thirst, tiredness, cramps, headache, nausea/ vomiting, fainting, prickly heat or urinogentital issues was 96% (*p* < 0.001). Within the organized sector, workplace WBGT > 27 °C was associated with significantly more heat-related symptoms (74%; chi square = 5.152, *p* = 0.023), as compared to workplaces where WBGT ≤ 27 °C. Workers with heavy workloads reported significantly more health issues (chi square = 23.67, *p* ≤ 0.001) compared to workers with perceived light and moderate workloads.

**Table 3:** Worker perceptions of heat-related health and productivity impacts resulting from occupational heat stress in various sectors in India.

Sl. No	Workplaces	Perceived Impacts on Health #, (%)	Perceived Impacts on Productivity #, (%)
**1.**	Agriculture (*n* = 23)	23 (100)	16 (70)
2	Auto parts manufacturing (*n* = 31)	31 (100)	17 (55)
3	Auto spare parts manufacturing (*n* = 10)	10 (100)	6 (60)
4	Auto manufacturing (*n* = 12)	10 (83)	7 (70)
5	Bakery (*n* = 27)	25 (93)	17 (63)
6	Brick manufacturing (22)	22 (100)	22 (100)
7	Building maintenance (*n* = 80)	75 (94)	41 (51)
8	Color Dye works (*n* = 4)	1 (25)	0
9	Construction (*n* = 52)	47 (90)	44 (85)
10	Die Casting (*n* = 11)	11 (100)	7 (64)
11	Heavy truck manufacturing (*n* = 12)	11(92)	7 (59)
12	Hotel (*n* = 19)	19 (100)	15 (79)
13	Laundry (*n* = 36)	34 (94)	14 (39)
14	Leather works (*n* = 12)	11 (92)	6 (50)
15	Metal fabrication (*n* = 21)	21 (100)	20 (95)
16	Security (*n* = 31)	31 (100)	28 (90)
17	Snack Preparation (*n* = 14)	14 (100)	6 (43)
18	Transport (*n* = 25)	25 (100)	16 (64)

*n* = number of workers interviewed in each workplace, # = number of workers reporting impacts.

Of the workers’ assessed in both hotter and cooler seasons, 65% *vs.* 32% were exposed to higher than recommended WBGT levels in hotter and cooler seasons respectively. This corresponded to a significant increase in self-reported heat-related health impacts (*p* ≤ 0.011) and productivity losses (*p* ≤ 0.016) as well as an increase of 1.8 **°**C–4.3 **°**C WBGT in the hotter season, as compared to the cooler.

### 3.4. Productivity Impacts

Overall, 57% (*Z* = 7.02, *p* < 0.0001) of the study population reported having experienced productivity losses due to heat stress. Of the 442 workers, approximately 62% reported reduced productivity by not achieving targets, 30% reported absenteeism as a reason for productivity loss and 25% workers’ reported lost wages due to fatigue/sickness due to workplace heat-stress. As illustrated in Table 3, productivity issues were reported most commonly among outdoor/semi outdoor occupations with high workload (e.g., brick manufacturing, metal fabrication, construction *etc.*). Loss of productivity was reported less frequently among indoor occupations. Productivity loss due to heat stress was similarly reported between organized and unorganized sectors. Workers with heavy workloads (77%) reported higher productivity losses which was statistically significant (chi square= 15.82, *p* ≤ 0.001) as compared to workers with light (54%) and moderate (60%) workloads. Multivariate logistic regression analysis revealed that males (OR = 1.881, *p* = 0.010) and workers with heavy workload (moderate workload OR = 0.393, *p* < 0.0001 & light workload OR = 0.336, *p* = 0.007) were significantly affected by heat-related productivity losses.

## 4. Discussion

### 4.1. Heat Stress Profile

Many workplaces were found to exceed the recommended heat TLVs for the observed work intensity in both hotter and cooler months. Workplaces that exceeded the recommended TLV involved processes with a high heat generation and/or were outdoor jobs, like agriculture, auto manufacturing, construction, brick manufacturing and die casting. In the current study, outdoor workers were exposed to higher heat levels in hotter periods (Figure 1). However, seasonal variations in heat levels were minimal for workplaces with high heat generating processes and many indoor workplaces had higher WBGTs than recorded outdoor ambient temperatures, irrespective of the season. This is presumably the result of heat generating processes and/or lack of proper ventilation. These findings agree with earlier findings from the automotive industry in India, indicating that heat stress is as yet still inadequately controlled in India’s organized sector [34]. This is concerning given that India has become the world’s fastest growing large economy and that this growth is anticipated to accelerate further [35]. The potential scale of workers at risk due to work-related heat stress is clearly evident from (Figure 1) which was also substantiated by the interviews with the workers, many of whom perceived occupational heat stress as having negative implications on their health and productivities (Table 3). Notably, heavy manual work was predominantly performed in the unorganized sector. It could be suggested that workers from the unorganized sector are more vulnerable to the ill-effects of heat stress as they are less likely to receive appropriate heat mitigation, protection or compensation for loss of production. However in the current study workers from both the organized and unorganized work sector were exposed to excessive levels of heat stress. Thus, it is important that heat-protection and mitigation policies consider both the organized and unorganized work sectors. Similarly high occupational heat stress profiles that exceed recommended TLVs have also been demonstrated in other studies conducted in India [28,34] and around the world [3,16,25]. From such evidence it can suggested that occupational heat-protection and mitigation requires more attention and action in many regions of the world.

### 4.2. Health Impacts (Heat Related Symptoms)

In tropical settings, high ambient temperatures and humidity levels significantly influence indoor temperatures, which when combined with heat generating processes exposes workers to potential health risks and productivity decrements [8,36]. Physical exertion combined with a lack of automation and cooling intervention exacerbates this heat stress [22,37]. In the current study, heat related symptoms and consequent potential health impacts were less pronounced in the cooler seasons, as perceived by the workers (Table 4). Interestingly, workplace WBGTs during hotter and cooler seasons were not largely different particularly during the heat of the day (increasing 1.8 **°**C–4.3 **°**C, Table 4). Yet the perceived impact of heat stress on workers’ health and productivity perceptions was significant. From this study and previous Indian-based research [28,34], it appears that modest increases in global temperature, as predicted with climate change, will have major impacts on workers in India and other similar tropical countries where workers are already exposed to excessively high heat levels.

**Table 4:** Repeat measures of workers (*n* = 72) perception on health and productivity losses during hotter and cooler seasons in Indian workplaces.

Season	Avg. WBGT Range (°C)	Workers Exceeding TLV	Health Impacts (%)	*p* Value	Productivity losses (%)	*p* Value
Hotter (April–June)	26.5–32.2	47	87	0.011	48	0.016
Cooler (November–January)	22.2–30.4	23	67		25	

TLV, Threshold Limit Values.

### 4.3. Productivity

Productivity loss of workers and consequent economic decrements is strongly dependent on thermal conditions, particularly when performing physically demanding work [38]. When a worker is working in very hot conditions, the natural response of the body is to slow down his/her physical activity in order to moderate internal body core temperature rising higher [18,27]. Due to this protective mechanism, in very hot environments work output is reduced leading to productivity losses. In an analysis of Thai industrial workers [16], ~60% of workers reported loss of working capacity in the heat, and approximately 20% were more vulnerable to heat illnesses during the hotter months. In the current study, over half of the interviewed workers reported productivity losses due to heat stress. It has previously been estimated that at above 27 °C, a one degree change in WBGT is associated with 4%–8% decline in productivity in South and Central Indian industries [20]. Given that in the cooler season 8 workplaces and in the hotter season 16 workplaces had a daily average WBGT above 27 °C these self-perceived rating of productivity may even underestimate the true heat-related productivity impact experience by workers. Productivity issues were reported most commonly among outdoor/semi outdoor occupations with high workload (e.g., brick manufacturing, metal fabrication, construction *etc.*), whereas productivity losses were reported less frequently among indoor workers (Table 3). This is presumably due to pre-existing cooling control measures, including some minimal level of automation to reduce physical exertion, in indoor workplaces. The productivity losses were perceived to be less in cooler seasons in the same workplaces, with workers performing the same work intensities and work profile (Table 4). This indicates that workers are aware of cooler temperatures and have a negative perception of how hotter temperatures affect their health and work productivity. In the face of increasing global and regional temperatures, it could be expected that a modest increases in temperature resulting from climate change could significantly impact the health and productivity of the exposed worker. Findings from the current study support this as workers perceptions of negative health consequences and productivity losses increased significantly with modest increases in workplace WBGT in hotter, as compares to the cooler season in southern India. This study is limited to the fact that the self-reported productivity losses may not be a true estimate but may be an overestimate or underestimate of the actual losses due to participant bias. However, non-invasive technologies have been successfully used to estimate the physical exertion of the workers working in heat and the influence of heat on physical exertion [39,40,41,42] which could be a much more accurate estimate of the productivity losses. Within the limitation of the study, the findings do concur with other studies that have also shown that changes in workplace temperature by even a few degrees can significantly influence the performance of a worker in several tasks including clerical work, factory work, signal recognition, time to respond to signals, learning performance, reading speed and comprehension, multiplication speed *etc.* [43,44,45]. Other detrimental consequences of excessive heat exposure include increased accident rates, reduced productivity and impaired physical and cognitive performance [18,46]. Our study results showed that workload was a significant predictor for developing heat-related health problems and workers with light workloads were less likely to develop problems related to heat strain than workers engaged in heavy work. Subsequently, reduction of hard manual labor and increased mechanization/automation can largely influence the heat-stress levels in a workplace. Additionally provision of cooling interventions will also greatly alleviate workers’ heat stress.

The importance of having a thermally comfortable workplaces to improve the health and productivity of the workers is influenced by numerous confounders including capital cost investment, management cooperation, worker behavior and “risk perception” towards heat-stress to name a few. Studies that estimate the local, regional, country and global-level impacts of economic losses due to heat stress [47] have thrown light on the importance of mitigating climate change with respect to protecting the health, safety and productivity of the future workforce.

## 5. Conclusions

This is the first study to investigate local heat exposure levels in Southern India (as described via WBGT) in a variety of organized and unorganized workplaces during both hotter and cooler months. The perceptions of the impact of heat stress from 442 workers were also evaluated. The novel findings from this study are as follows: (1) workers in India are subjected to high heat conditions in their workplaces irrespective of the season; and (2) occupational heat stress had negative implications on health and productivities of exposed workers. The current study provides clear evidence to re-emphasize that heat stress is a significant occupational health risk in tropical settings like India, especially in the context of climate change. Interventions to tackle this issue will benefit industries and workers in multiple ways such as improving their health, productivity, and economy. With the threat of climate change and increasing temperatures the detrimental impacts of heat stress on working population can be expected to also increase. It is imperative to include cost-effective, feasible and sustainable measures to control workplace heat stress. This study can be a forerunner for further conducting in-depth research in this area and eventually provide insights into improvement of guidelines, safety and health standards for workers in the context of climate changes and related health impacts. Further studies to identify signs and symptoms of excessive heat stress are needed for high heat exposure sectors. Studies, like the one presented, provide data to model future risks of workplace heat stress so that strong protective policies, climate change adaptation and mitigation measures can be rolled out to protect the present and future workforce.

## Supplementary Files

Supplementary File 1
