# Supplementary material for: Occupational Heat Stress Profiles in Selected Workplaces in India
Source: Int J Environ Res Public Health. 2015 Dec 29;13(1):89. doi: 10.3390/ijerph13010089 (PMC4730480; doi:10.3390/ijerph13010089)
Supplement: Supplementary File 1 [file ijerph-13-00089-s001.pdf]

International Journal of *Environmental Research and Public Health*

MDPI

Supplementary Information

Occupational Heat Stress Profiles in Selected Workplaces in India

Main Study Questionnaire

Part 1 General Information about Person Interviewed and the Organization She/He Represents

1. Name:
2. ID No:
3. Date of interview:
4. Name of the interviewer:
5. Name of the industry:
6. Location of the industry:
7. Type of industry:
8. Age:
9. Age group: □1 10–20/□2 21–30/□3 31–40/□4 41–50/□5 51–60/□6 61–70
10. Sex: □1 Male/□2 Female
11. Education: □1 Illiterate/□2 Primary/□3 Secondary/□4 Higher Secondary/□5 University/ □6 Polytechnic/Diploma
12. Designation: □1 Worker/□2 Supervisory/□3 Manager
13. Worker category:
14. Smoking: □1 Smoker/□2 Non Smoker/□3 Ex smoker
15. Consuming alcohol: □1 Yes/□2 No/□3 Ex
16. Any existing illness: □1 Diabetes/□2 Hypertension/□3 Respiratory illness, specify/□4 Others, specify/ □5 None

Part 2 Questions Concerning the Type of Work

1. Type of work: □1 Light/□2 Moderate/□3 Heavy/□4 Very Heavy
2. What was your previous job and where (relating to temp)? ____________
3. How long you are employed here? ____________ years/months (more than 6 months means acclimatized)
4. How many hours per day do you usually work excluding regular break timings? _________
5. Do you work near a direct heat source(naked flame/hot air/outdoors/radiant heat) □1 Yes/□2 No
6. Is the place you work well-ventilated? □1 Yes/□2 No
7. Do you have additional breaks during summer? □1 Yes/□2 No
    a. If yes, mention no. of hours/minutes_________________

Part 3 Questions in Relation to Heat Exposure at Work

1. Are you comfortable with the ambient temperature? □1 Yes/□2 No
2. Is heat exposure a problem during the hot season? □1 Yes/□2 No
3. How many months do you feel hot /uncomfortably hot in this workplace? □1 1–3 months/□2 4–6 months/□3 7–9 months/□4 9–12 months/□5 Never
4. Describe how bad the heat stress can be in the hot season. □1 Extremely bad/□2 Very bad/ □3 Bad/□4 Manageable/□5 No stress at all

Part 4 Questions Concerning Impacts of Heat on Health

1. Have you ever had these symptoms at work?

   a. Excessive sweating ☐1 Yes/☐2 No
   b. Muscle/Heat cramps ☐1 Yes/☐2 No
   c. Thirst ☐1 Yes/☐2 No
   d. Tiredness/weakness/☐1 Yes/☐2 No
   e. dizziness☐1 Yes/☐2 No
   f. headache ☐1 Yes/☐2 No
   g. nausea or vomiting☐1 Yes/☐2 No
   h. fainting☐1 Yes/☐2 No
   i. Prickly heat ☐1 Yes/☐2 No
   j. Heat stroke ☐1 Yes/☐2 No
   k. Others ____________________________

Part 5 Questions Concerning Impacts of Heat on Worker's Productivity

1. Have you ever taken sick leave/permission due to heat? ☐1 Yes/☐2 No

   a. If yes, approx. how many hours/days in a week/month? __________________

2. Have you lost any wages due to absenteeism in summer months? ☐1 Yes/☐2 No

   a. If yes, how much ____________________________? (currency)

3. Have you ever been advised/ever been admitted in hospital/medical centre to take off due to heat related illness? ☐1 Yes/☐2 No

   a. If yes, approximately how many days ____________________________

4. How does heat affect other aspects of your work (during hot seasons)
   ☐1 Absenteeism/☐2 Less productivity/☐3 Irritation/Interpersonal issues/Work related issues with manager/☐4 Take more time to complete same task
5. Do you have production target? ☐1 Yes/☐2 No

   a. If Yes, how much? ____________________________

6. Do you complete your production target? ☐1 Yes/☐2 No

   a. If no, how much target is not completed? ____________________________units

7. To achieve production target or complete work do you have to work extra hours?
   ☐1 Yes/☐2 No

   a. If yes, how many extra hours ____________________________

Part 6 Questions Concerning Impacts of Clothing on Heat Stress and Productivity

1. Dress material of the workers (Indian equivalent to ACGIH)
   ☐1 Breathable cotton/☐2 Thick cotton overall/☐3 Rayon/Nylon/
   ☐4 Plastic PPE/☐5 Others ________________________
2. Worker perception about dress Material of the worker:
   ☐1Comfortable/☐2 Moderately comfortable/☐3 Uncomfortable/☐4 Others _____________
3. If uncomfortable, can you give any suggestions to improve? ______________________
4. Do you feel hotter with uniform/Does the Dress Material increase heat stress/comfort?
   ☐1 Sure/☐2 Maybe/☐3 Not sure/☐4 No, not at all
5. Does clothing reduce your work output?
   ☐1 Sure/☐2 Maybe/☐3 Not sure/☐4 No, not at all

Part 7 Questions Concerning Coping Mechanisms

1. How do you limit heat exposure/cope with heat exhaustion, when needed?
   □1 take rest/□2 Change/remove clothing/□3 drink water/□4 cool shower, bath, or sponge bath/□5 move to an air-conditioned/cooler environment/□6 any other method, do specify ____________________
2. Is sufficient water available at all times when you need it? □1 Yes/□2 No
3. Do you drink water at work? □1 Yes/□2 No
   b. If yes, how much?____________________
4. Do you take any traditional special diet to cope with heat? □1 Yes/□2 No
5. What traditional or other methods do you adopt for coping with heat?
   ____
6. Do you spend more money during hot seasons to cope with heat? □1 Yes/□2 No
   a If yes, specify ____________________ (Currency units)
7. Do you spend more time to cope with heat? □1 Yes/□2 No
   a. If yes how much _________________ (min/h)—convert to % of productivity time or personal time
8. Does the time spent on coping heat impact your social life? □1 Yes/□2 No
9. How does it affect your social life? □1 Moderately/□2 Highly/□3 Extremely/□4 No impact

Part 8 Questions Concerning Access to Toilets

1 Do you have access to toilets at work? □1 Yes/□2 No

Part 9 Questions If You Have Access to Toilets

1. Do you use the toilets? □1 Yes/□2 No
   a. If yes: How often
      a. 1–2 times per day
      b. 3–4 times per day
      c. 5–6 times per day
      d. >6 times per day
   b. If no: Why not
      a. They are too far away
      b. Difficulties to get away from work
      c. They are to dirty
      d. Others
2. Can you talk about it in the open? □1 Yes/□2 No
   a. If no: Why not
      a. It makes you feel uncomfortable
      b. It is taboo
      c. People around you would treat you outrageous
      d. Others

Part 10 Questions If You Don't Have Access to Toilets

1. How often do you go to urinate and defecate?
   a. 0 times
   b. 1–2 times

c. 3–4 times
d. 5–6 times
e. >6 times

If b–e:

2. Where do you go to urinate and defecate?
    a. An hidden area (small building *etc.*)
    b. In the forest
    c. On the streets
    d. Others
3. Do you go alone? ☐1 Yes/☐2 No
4. Do you feel comfortable with that option? ☐1 Yes/☐2 No
    a. If no: Why not?
        a. Feel scared
        b. Needs to hold it for longer than convenient
        c. Feel uncomfortable
        d. Others
5. Have something inconvenient happened to you when you go for urination or defecation? ☐1 Yes/☐2 No
    a. If yes: What have happened
        a. Bitten by an animal
        b. Someone has been watching
        c. Assaulted
        d. Others
6. Do you feel that you have to hold it for longer time than convenient? ☐1 Yes/☐2 No

a. If yes: For how long approximately?
    a. 1–2 h
    b. 3–4 h
    c. 4–5 h
    d. >6 h

7. Do you sometimes have to stay home from work because of the toilet situation during some stomach infection or similar situation? ☐1 Yes/☐2 No
    a. If yes: How often
        a. Once per year
        b. 2–5 times per year
        c. 6–11 times per year
        d. Once per month
        e. More than 1 time per month
8. Do you think your working situation would be better if you had access to toilets? ☐1 Yes/☐2 No

Part 11 Questions Concerning Reproduction History and Menstrual History

1. Is your menstrual cycle regular? ☐1 Yes/☐2 No/ ☐3 Others
    a. If yes: Can you work during your menstrual cycle? ☐1 Yes/☐2 No/☐3 Others

b. If no: Why not?

   a. Nowhere to take care of sanitary pads
   b. Feel to dirty
   c. Not allowed
   d. Lack of toilets
   e. Others

2. Do you have access to sanitary pads during your menstrual cycle?
   ☐1 Yes/☐2 No
3. Do you have access to a place to change sanitary pads during your menstrual cycle?
   ☐1 Yes/☐2 No

   a. If yes: Do you go there? ☐1 Yes/☐2 No
   b. If no: Why not

      a. It is too far away
      b. It is to dirty
      c. It is not private enough
      d. Afraid that someone will be aware of you menstrual cycle
      e. Others

4. If you don't have access: How do you manage?

   a. Go somewhere else
   b. Wait the whole day
   c. Stay home from work
   d. Others

Part 12 Questions Concerning Access to Drinking Water

1. Do you have access to drinking water at your work? ☐1 Yes/☐2 No

   a. If yes: From where do you get that water?

      a. From a water tank
      b. Water bottles
      c. A river or lake *etc.* close to the area
      d. Others

2. Do you drink when you feel thirst? ☐1 Yes/☐2 No

   a. If no: why not?

      a. Not access to water
      b. It will be to difficult to hold it
      c. The work do not allow it
      d. Others

3. Do you think you would drink more if you had better access to toilets? ☐1 Yes/☐2 No

   a. If no: Why not?

      a. The access to water is not good
      b. The work does not allow it
      c. Others

Part 13 Questions Concerning Effects on Daily Life

1. Does the toilet situation impact your social life? ☐1 Yes/☐2 No

a. If yes: How?

a. Have to stay home more than if there had been toilets
b. Feeling tired
c. Others

Part 14 Questions Concerning Urinary Tract Infection

1. Do you have burning sensation during urination? □1 Yes/□2 No
   a. If yes: How often?
      a. Every day
      b. Few days a week
      c. Once a week
      d. Less than once a week
   b. If yes: For how long?
      a. The last week
      b. The last month
      c. The last 6 months
      d. Longer than 6 months
2. Do you have difficulties to hold it? □1 Yes/□2 No
3. Do you feel difficulty while urinating even if it is needed? □1 Yes/□2 No
   a. If yes: How often?
      a. The last week
      b. The last month
      c. The last 6 months
      d. Longer than 6 months
4. Do you think this problem would have been better if you had better access to toilets? □1 Yes/□2 No
5. Have you ever been admitted to hospital/medical centre due to urinal or defecation problems? □1 Yes/□2 No
   a. If yes: Approximately for how many days? ______________

Part 15 Questions Concerning Kidney Problems

2. Have you noticed changes in your urine volume? □1 Yes/□2 No
3. Do you have excessive tiredness or skin itching? □1 Yes/□2 No
4. Do you have numbness or swollen legs or hands due to water retention? □1 Yes/□2 No
   a. If Yes to 1,2 and 3, What is the color of your urine?
      (a) Reddish
      (b) Dark Yellow
      (c) Yellow
      (d) Colorless
      (e) Have not noticed
      (f) Don't know
5. Have you been treated for kidney stones? □1 Yes/□2 No
   a. If yes: When? ______________
6. Do you feel pain in the bottom of your back? □1 Yes/□2 No

a. If yes: For how long?
(a) The last week
(b) The last month
(c) The last 2–6 month
(d) Longer than the last 6 months

7. Do you think this problem would have been better if you had better access to toilets?
☐1 Yes/☐2 No
8. Have you ever been admitted to hospital/medical centre because of kidney problems?
☐1 Yes/☐2 No
a. If yes: Approximately for how many days? ______________

Part 16 Questions concerning dehydration

1. Do you feel dehydrated? ☐1 Yes/☐2 No
2. Do you have any pressure ulcers? ☐1 Yes/☐2 No
3. Do you have any other skin conditions? ☐1 Yes/☐2 No
4. Do you have nausea or fainting spells? ☐1 Yes/☐2 No
5. Have you ever been admitted to hospital/medical centre due to dehydration?
☐1 Yes/☐2 No
a. If yes: Approximately for how many days? ______________
6. Do you think this problem would have been better if you had better access to toilets?
☐1 Yes/☐2 No

Part 17 Questions Concerning Drinking Defecated Water

1. Do you feel constant hunger even if you are eating? ☐1 Yes/☐2 No
2. Do you have diarrhea? ☐1 Yes/☐2 No
3. Do you have diarrhea with blood and mucus? ☐1 Yes/☐2 No
4. Do you feel swollen in your stomach? ☐1 Yes/☐2 No
a. If yes: Which part?
(a) Upper part
(b) Lower part
(c) The whole belly
5. Do you feel itching around anus? ☐1 Yes/☐2 No
6. Do you feel cramps in your stomach? ☐1 Yes/☐2 No
7. Do you have fever? ☐1 Yes/☐2 No
a. If yes: How often
(a) Every day
(b) 1–6 days per week
(c) A few days per month
(d) A few days per year
(e) Less than a few days per year
8. Do you think this problem would have been better if you had better access to toilets?
☐1 Yes/☐2 No
9. Have you ever been admitted to hospital/medical entre because of defecation problems?
☐1 Yes/☐2 No
a. If yes: Approximately for how many days? ______________

Part 18 Questions Concerning Menstrual Cycle

1. Do you wash yourself regularly during your menstrual cycle? ☐1 Yes/☐2 No
2. Do you feel itching around your genitals? ☐1 Yes/☐2 No

a. If No, Do you think this problem would have been better if you had better access to toilets? □1 Yes/□2 No

Part 19 Questions Concerning Other Health Effects

1. Do you feel any other health problems than the ones mentioned above? □1 Yes/□2 No
   a. If yes: What kind of health problems?

   ____

   b. If yes: Do you think they may be caused by lack of toilets? □1 Yes/□2 No
2. Do you think it would be less health problems if you would drink more? □1 Yes/□2 No

© 2015 by the authors; licensee MDPI, Basel, Switzerland. This article is an open access article distributed under the terms and conditions of the Creative Commons by Attribution (CC-BY) license (http://creativecommons.org/licenses/by/4.0/).
